# The Association Between Longitudinal Changes in Body Mass Index and Longitudinal Changes in Hours of Screen Time, and Hours of Physical Activity in German Children

**DOI:** 10.1002/osp4.70031

**Published:** 2024-12-24

**Authors:** Sophie Hoehne, Olga Pollatos, Petra Warschburger, Daniel Zimprich

**Affiliations:** ^1^ Department of Developmental Psychology Institute of Psychology and Education Ulm University Ulm Germany; ^2^ Department of Clinical & Health Psychology Institute of Psychology and Education Ulm University Ulm Germany; ^3^ Department of Psychology University of Potsdam Potsdam Germany

**Keywords:** body weight, childhood, leisure activities, obesity, overweight, television viewing

## Abstract

**Introduction:**

The association of screen time and physical activity with body weight in children has been investigated in cross‐sectional and prospective studies, as well as randomized controlled trials. The present study extends previous research by examining how longitudinal within‐person changes in screen time and physical activity relate to changes in Body Mass Index (BMI) in children, and how changes in screen time and physical activity relate to each other.

**Methods:**

The data for the present study came from the PIER Youth Study. Data were collected from 971 children and their parents at two time points approximately 1 year apart. A multilevel modeling approach with measurement occasions nested within children was used to model changes in BMI across age.

**Results:**

Within a child, a change in daily hours of TV viewing was associated with a corresponding change in BMI, whereas a change in daily hours of physical activity was associated with an opposite change in BMI. Within‐person correlations between changes in screen time and physical activity were small but positive.

**Conclusion:**

The present findings have important implications for interventions to reduce and prevent childhood overweight and obesity. Specifically, interventions should focus on both reducing daily TV viewing and promoting physical activity.

## Graphical abstract

The association of changes in screen time and changes in physical activity with changes in body weight over the course of 1 year was investigated in German children. Over the course of 1 year, a change in daily hours of TV viewing was associated with a corresponding change in BMI in German children aged 6–11 years. Moreover, over the course of 1 year, a change in daily hours of physical activity was associated with an opposite change in BMI in German children aged 6–11 years.

## 1 Introduction

According to the World Health Organization, in 2016, 39 million children under the age of five and 340 million children and adolescents between the ages of five and 19 were overweight [1]. In Europe, data for 2018 to 2020 show that, overall, 29% of children aged seven to nine were overweight and 12% had obesity. Specifically, the prevalence rate in Germany from 2018–2020 was 25.9% for overweight and 10.3% for obesity among 5‐ to 9‐year‐olds [2].

The importance of childhood for overweight and obesity stems from the fact that it represents a major risk factor for overweight and obesity also during adulthood and the resulting health problems [3]. Moreover, health risks such as glucose intolerance, high blood pressure, sleep apnea, asthma, depression, and hyperlipidemia can already manifest in childhood and adolescence [4], emphasizing the negative role of childhood overweight and obesity.

A major risk factor for the development of obesity during childhood and adolescence is an increase in sedentary behavior, that is, behavior that requires a low expenditure of energy and that takes place in a sitting or lying position [5, 6, 7]. The reverse pattern has also been shown, with children with obesity who decreased sedentary behavior and increased physical activity showing reductions in both body fat and percentage overweight [8, 9]. Typical examples of sedentary behaviors include reading, quiet playing, watching TV, playing video games, and the use of computers or other electronic devices. Importantly, the last 2 decades have seen a dramatic increase in access to Internet services and digital technologies, so that to date screen time, particularly playing video games and watching television, appears to have become the most common form of sedentary leisure time behavior among children and adolescents [10, 11]. Expert groups have proposed guidelines for maximum screen time for children. For instance, in 2016, the American Academy of Pediatrics suggested limiting daily screen time to no more than an hour for children aged 2–5, and that parents should agree to limit screen time for children aged 6 years and older [12]. Similar guidelines have been proposed by the Canadian Pediatric Society [13]. However, a study of screen time among 9‐ to 18‐year‐old children and adolescents in Germany between 2019 and 2020 found that only about 35% of all children and adolescents met the suggested guidelines for screen time [14], while 65% exceeded the recommended maximum.

In relation to obesity, a variety of studies have shown that children and adolescents who spend more time in front of screens are more likely to be overweight or obese. In particular, one review found that of 40 studies of weight status and screen time, mainly conducted in the United States and Europe but also in other countries such as Brazil and Saudi Arabia, 85% showed a positive association between obesity and screen time in 5‐ to 19‐year‐olds [11]. The evidence came from different types of studies, but most were observational, cross‐sectional studies. Moreover, in a review of only cross‐sectional studies conducted in either the United States or other developed countries [15], the majority of studies found a positive association between screen time and body weight in children and adolescents aged 2–19 years. Another study reported that more screen time was associated with poor weight status in the data from 12 different countries, demonstrating that this association appears to be relatively global [16]. Cross‐sectional studies are a first step in establishing associations between sedentary behavior and obesity, but longitudinal studies can be used to determine the directionality of the effects.

Although fewer in number than cross‐sectional studies, there are also a number of longitudinal prospective studies that report analogous associations between screen time and overweight and obesity in children and adolescents. For instance, among white girls in the United States, the time spent watching TV at age 10 was associated with an increase in body mass index (BMI) over 4 years [17]. Similarly, among 3‐year‐old Canadian children followed for 2 years, those who had more than 1 h of screen‐time per day at age three were more likely to have overweight or obesity at age five [18].

Finally, a few randomized controlled trials have tested the effects of experimentally induced reductions in screen time on children's body weight. The results are mixed. A review of studies of children up to 13 years of age, conducted mainly in the United States but also in other developed countries, found that interventions to reduce screen time were associated with an increase in physical activity and weight‐related outcomes compared to control groups [9]. However, in a recent meta‐analysis of randomized controlled trials of screen time interventions on BMI in children and adolescents between the ages of 3–15 years from the United States, New Zealand, Türkiye, Canada, and Australia, interventions were only effective in reducing screen time, but had no effect on body weight [19]. Therefore, it is not yet clear whether reducing or increasing screen time is related to changes in body weight in children and adolescents and can therefore be used as a target area for prevention.

Importantly, the effects of screen time on body weight may work through different mechanisms, such as unhealthy eating and snack consumption during screen time, as well as a reduction in physical activity [20]. In terms of unhealthy eating, screen time, with a particular focus on TV viewing, has been found to influence food preferences and food intake in children and adolescents [11]. Specifically, TV viewing is associated with advertisements for energy‐dense foods, and also with reduced awareness during eating and increased craving for snacks, all of which can influence body weight [21]. Moreover, increases in screen time have been associated with a reduction in physical activity [22]. It might be expected that the association between sedentary behavior and increased body weight would also hold in the opposite direction, in the sense that more physically active children would have fewer risks of being overweight. In fact, a series of studies demonstrated that higher engagement in physical activity was associated with an improved weight status in children [23] and adolescents [24]. Although increased screen time has been linked to reduced physical activity [22], it has been found that a reduction in screen time does not necessarily lead to an increase in physical activity [25]. Therefore, when examining the impact of changes in screen time on body weight, it is also necessary to examine changes in physical activity and its impact on body weight.

The present study sought to examine how naturally occurring changes in screen time were related to changes in children's BMI over the course of approximately 1 year in an observational setting. To the best of our knowledge, no study to date has examined within‐person correlations of change, that is, how a change in screen time is related to a change in BMI in the same child, which represents an alternative, relatively strong test of the association [15]. The first hypothesis of the present study was therefore that there would be a positive within‐person correlation between changes in children's screen time and changes in children's BMI. In addition to changes in children's screen time, the present study also included changes in children's physical activity. Therefore, the second hypothesis of the present study was that there would be a negative within‐person correlation between changes in children's physical activity and changes in children's BMI. Finally, the present study aimed to investigate how a within‐person change in children's screen time was related to a within‐person change in children's physical activity.

## 2 Methods

### 2.1 Sample

The data for the present study come from the PIER Youth Study (see http://www.uni‐potsdam.de/pier‐studie), a large longitudinal study of intrapersonal developmental risk factors in childhood and adolescence that began in 2012 [26]. The PIER data have already been used in other studies, but these have focused on different research questions and therefore different aspects of the data [27, 28, 29, 30, 31]. The present data include two assessments approximately 1 year apart (*M* = 273 days, SD = 55 days). At the first assessment (T1), 1657 children aged 6 to 11 (52.1% girls) and their parents (*N* = 1339) were recruited from 33 elementary schools in the German state of Brandenburg. Of these children, now aged 7 to 11, 1619 took part in the second assessment (T2), as did 1160 parents. The schools were selected to represent a range of rural and urban social backgrounds. The present analysis uses data from 971 children (51.7% girls) at T1 (*M* age = 8.30 years, SD = 0.93) and T2 (*M* age = 9.04 years, SD = 0.91) and their parents for whom complete measures of the constructs relevant to the present study were available.

A comparison of the 971 children included in the analyses of this study with the 687 children not included due to missing values showed a significantly lower average BMI at T1 in the former group (*t* = 2.49, *df* = 1656, *p* < 0.013), but the effect size was negligible (*R* ^2^ = 0.4%). In addition, the included children came from families with a significantly higher level of education (*t* = 6.70, *df* = 1656, *p* < 0.001) and a significantly higher job status (*t* = 3.60, *df* = 1653, *p* < 0.002, *R* ^2^ = 0.9%). With respect to parents' education, the difference amounted to a small to medium effect (*R* ^2^ = 3.2%). No significant differences were found for leisure activities.

### 2.2 Procedure

Each child's primary caregiver signed an informed consent form prior to participation. The children and their parents were informed about the study procedures and aims and were assured of their privacy. On both measurement occasions, T1 and T2, children underwent two assessments about 7 days apart. Each assessment lasted 50 min. The tests were administered by a trained and supervised doctoral student or research assistant in a quiet room during the morning hours, either at home or at school. At both T1 and T2, parents were given additional questions to answer either in printed form or online. Most information was provided by the mother (T1: 69%, T2: 76%) or both parents (T1: 22%, T2: 16%). Children were given a cinema voucher at T1 and T2 in exchange for their participation. The study has been approved by the Research Ethics Board at the University of Potsdam and the Ministry of Education, Youth and Sports of the Federal State of Brandenburg.

### 2.3 Measures

#### 2.3.1 Body Mass Index and Weight Status

Each child's body weight and height were measured using calibrated ultrasound measurement devices and calibrated digital scales while wearing light clothing without heavy jackets, hats, or shoes. The ratio of weight in kilograms divided by the square of height in meters was used to calculate BMI. Classifications of overweight and obesity were adjusted for sex and age using national reference data from German children [32].

In many studies, instead of the “raw” BMI scores, the BMI‐SDS or BMI‐z scores, based on a comparison with norm data categorized by age brackets and sex, are used [32]. For the present research, this appears to be problematic for two reasons. First, the German norm data are already 11 years old and, moreover, the age brackets are relatively broad (comprising 6 months instead of, e.g., the US data, where 1‐month intervals are used). Second, the transformation of “raw” BMI scores into standardized BMI scores (i.e., SDS or z), although linear within every age bracket and sex combination, is non‐linear across age brackets, which implies that the same amount of BMI change across time is weighted differentially depending on which age bracket a child belongs to at T1 and T2. This is problematic in the current study because BMI changes are modeled within the same child. Moreover, given the longitudinal design, some children move up one age bracket from T1 to T2, while others move up two age brackets, which further limits the comparability of results between children. Therefore, we decided to use the “raw” BMI scores in the present analysis and statistically control for the effects of sex and age.

Children who were above the 90^th^ BMI percentile for their sex and age group were classified as overweight. At T1, 65 children (36 girls) were overweight without having obesity, whereas at T2, 74 children (35 girls) were overweight without having obesity. Similarly, children who were above the 97th BMI percentile for their sex and age group were classified as having obesity. At T1, 43 children (24 girls) had obesity and at T2, 40 children (23 girls) had obesity. According to International Obesity Task Force (IOTF) standards [33], at T1, 109 children had overweight (64 girls) and 37 had obesity (21 girls), whereas at T2, 123 children had overweight (63 girls) and 34 had obesity (20 girls). Furthermore, using WHO standards [34], at T1, 149 children had overweight (79 girls) and 73 had obesity (35 girls), whereas at T2, 172 children had overweight (83 girls) and 73 had obesity (33 girls).

#### 2.3.2 Socioeconomic Status

The highest level of parental education was used as a measure of socio‐economic status. Parents indicated their formal education on a scale from 1 (“none”) to 6 (“university degree”). Moreover, parents' highest job level was used as a second indicator of socioeconomic status using the Blossfeld job classifications [35] on a scale from 0 (lowest job level) to 4 (highest job level).

#### 2.3.3 Leisure Activities

Three different leisure time activities were measured: (1) daily hours of watching TV, including DVD and video tapes, (2) daily hours of using computers, including surfing the Internet, and (3) daily hours of physical activity. Items were adapted from the KiGGS‐study (cross‐sectional and longitudinal study on child and adolescent health in Germany) [36]. Parents reported the amount of time their child spent each day in the three different activities. Questions were answered on a five‐point Likert type scale (0 = “no time at all,” 1 = “approximately 30 min,” 2 = “1–2 h,” 3 = “3–4 h,” 4 = “more than 4 h”). Because category 4 was extremely rare, categories 3 and 4 were collapsed, resulting in three variables with four categories each (see Table 1).

**TABLE 1:** Demographic information by categories of physical activity, TV viewing, and computer/Internet time at first assessment (T1).

	*N* (%)	Mean BMI^a^	Percentage overweight			Percentage obese		
			German reference data^b^	IOTF^c^	WHO^d^	German reference data^b^	IOTF^c^	WHO^d^
Sex								
Male	469 (48.3)	16.79	6.2	9.6	14.9	4.0	3.4	8.1
Female	502 (51.7)	16.82	7.2	12.7	15.7	4.8	4.2	7.0
Parental education								
1 (Hauptschule)	80 (8.2)	18.09	16.2	22.5	20.0	13.7	11.2	20.0
1 (Realschule)	383 (39.4)	17.09	7.8	14.6	18.0	5.5	4.7	9.1
2 (Gymnasium)	136 (14.0)	16.62	3.7	5.9	13.2	4.4	4.4	6.6
3 (Studium) New York	372 (38.3)	16.30	4.6	7.3	12.4	1.3	1.1	3.5
Parental job status								
0	43 (4.4)	17.85	11.6	18.6	27.9	9.3	9.3	11.6
1	105 (10.8)	17.73	12.4	20.0	24.8	12.4	11.4	18.1
2	63 (6.5)	16.81	4.8	11.1	19.0	4.8	4.8	6.3
3	490 (50.5)	16.79	6.7	11.2	14.1	4.3	3.5	7.5
4	270 (27.8)	16.29	4.1	6.7	11.1	0.7	0.4	3.0
Hours of physical activity								
0	10 (1.0)	19.02	0.0	10.0	10.0	20.0	20.0	20.0
0–1	114 (11.7)	17.00	10.5	14.0	17.5	7.9	7.0	9.6
1–3	459 (47.3)	16.87	6.5	12.0	17.6	3.5	2.8	7.2
> 3	388 (40.0)	16.61	5.9	9.5	12.1	4.1	3.6	7.0
Hours of TV/DVD								
0	30 (3.1)	16.28	3.3	6.7	16.7	0.0	0.0	0.0
0–1	328 (33.8)	16.41	5.5	9.1	16.1	0.9	0.9	1.8
1–3	472 (48.6)	16.93	7.0	11.9	16.7	4.9	4.2	8.7
> 3	141 (14.5)	17.42	9.2	14.9	8.5	12.1	9.9	18.4
Hours of computer/Intern.								
0	378 (38.9)	16.82	5.0	10.0	18.2	4.5	4.2	5.8
0–1	414 (42.6)	16.63	8.0	12.3	14.7	2.7	1.9	6.8
1–3	165 (17.0)	17.15	7.9	12.1	11.5	7.3	6.1	12.1
> 3	14 (1.4)	17.47	0.0	0.0	0.0	21.4	21.4	21.4

^a^ *Differences BMI*: Sex: *t*(969) = −0.17 (*p* < 0.86), *d* = 0.01. Parental education: *F*(3, 967) = 14.72, *p* < 0.001, *R* ^2^ = 4.4%. Parental job status: *F*(4, 966) = 8.66, *p* < 0.001, *R* ^2^ = 3.5%. Physical activity: *F*(3, 967) = 3.76, *p* < 0.01, *R* ^2^ = 1.1%. TV/DVD: *F*(3, 967) = 6.57, *p* < 0.001, *R* ^2^ = 2.0%. Computer/Internet: *F*(3, 967) = 2.09, *p* = 0.10, *R* ^2^ = 0.6%.

^b^ *Percentage differences normal (with underweight)/overweight/obese: German reference data* [32]: Sex: *χ* ^2^ (2) = 0.72, *p* = 0.70, *w* = 0.03 (small effect). Parental education: *χ* ^2^ (6) = 45.35, *p* < 0.001, *w* = 0.22 (medium effect). Parental job status: *χ* ^2^ (8) = 39.28, *p* < 0.001, *w* = 0.20 (medium effect). Physical activity: *χ* ^2^ (6) = 13.98, *p* < 0.05, *w* = 0.12 (small effect). TV/DVD: *χ* ^2^ (6) = 34.53, *p* < 0.001, *w* = 0.19 (small to medium effect). Computer/Internet: *χ* ^2^ (6) = 19.54, *p* < 0.01, *w* = 0.14 (small to medium effect).

^c^ *Percentage differences normal (with underweight)/overweight/obese: IOTF* [33]: Sex: *χ* ^2^ (2) = 2.97, *p* = 0.23, *w* = 0.05 (small effect). Parental education: *χ* ^2^ (6) = 47.97, *p* < 0.001, *w* = 0.22 (medium effect). Parental job status: *χ* ^2^ (8) = 48.57, *p* < 0.001, *w* = 0.22 (medium effect). Physical activity: *χ* ^2^ (6) = 14.10, *p* < 0.05, *w* = 0.12 (small effect). TV/DVD: *χ* ^2^ (6) = 28.98, *p* < 0.001, *w* = 0.17 (small to medium effect). Computer/Internet: *χ* ^2^ (6) = 20.51, *p* < 0.01, *w* = 0.14 (small to medium effect).

^d^ *Percentage differences normal (with underweight)/overweight/obese: WHO* [34]: Sex: *χ* ^2^ (2) = 0.52, *p* = 0.77, *w* = 0.02 (small effect). Parental education: *χ* ^2^ (6) = 38.01, *p* < 0.001, *w* = 0.20 (medium effect). Parental job status: *χ* ^2^ (8) = 48.72, *p* < 0.001, *w* = 0.22 (medium effect). Physical activity: *χ* ^2^ (6) = 9.06, *p* = 0.17, *w* = 0.10 (small effect). TV/DVD: *χ* ^2^ (6) = 46.53, *p* < 0.001, *w* = 0.22 (medium effect). Computer/Internet: *χ* ^2^ (6) = 16.12, *p* < 0.05, *w* = 0.13 (small to medium effect).

### 2.4 Modeling Approach

In order to model changes in BMI across age groups, we used a multilevel model approach, with measurement occasions nested within children [37, 38]. However, compared to more typical parameterizations of multilevel models, we aimed to clearly distinguish between *inter*individual differences and *intra*individual change. In longitudinal data, one may distinguish between two types of effects, namely between‐person effects (cross‐sectional) and within‐person effects (longitudinal) [39, 40]. For example, children with a higher BMI may watch more TV regardless of the measurement occasion, which represents a cross‐sectional or between‐person effect. By contrast, children who increase in BMI across time more than other children may also increase their TV viewing hours more than other children do over time, which represents a longitudinal or within‐person effect. The second type of effect, that is, the within‐person effect, allows for stronger conclusions regarding the association between TV watching and BMI because it represents a true longitudinal effect [15]. To separate *within‐* and *between‐person* effects of a time‐varying predictor variable (e.g., hours of watching TV) on an outcome variable that changes over time (e.g., BMI), one can include both a person's average of the predictor variable and the person‐specific time‐varying deviation of the predictor variable from its average.

Predictor and control variables at Level 2, that is, individual means of physical activity, TV viewing and computer use, as well as age, sex, parental education and parental job status, were entered into the models grand‐mean centered, while predictors at Level 1, that is daily hours of TV consumption, computer use and physical activity at each measurement occasion, were entered group‐mean centered. As measures of effect size, we report Cohen's *d* for mean comparisons and Cohen's *w* for cross‐tabulations [41].

## 3 Results

Table 1 shows descriptive analyses of the children's BMI data at the first measurement occasion (T1). As can be seen from Table 1 (differences BMI), there were no sex differences in mean BMI. As expected, parental education was associated with children's BMI, with lower parental education being related to a higher average BMI. Similarly, lower parental job status was also related to a higher average BMI in the child. Pertaining to the activity measures, children with more hours of daily physical activity had a lower average BMI, whereas children with more hours of daily TV viewing had a higher average BMI. There were no differences in BMI for hours of computer or Internet use. In general, the effect sizes were in the small range. When the data are grouped according to weight status, similar differences emerge (see Table 1: percentage differences normal [including underweight]/overweight/obese). The percentage of children with overweight and obesity was significantly higher among children whose parents were less educated or had a lower job status. There was also an increased risk of being overweight or obese among children who were less physically active and those who watched more TV and spent more time using computers or the Internet. With regard to weight status grouping, effect sizes were in the small to medium range.

Estimates from Model 0 are shown in Table 2. As shown in Table 2, the cross‐sectional BMI for a boy of average age was estimated to be 16.81, and the BMI for a girl of average age was almost the same (mean BMI = 16.85). With each year of age, cross‐sectionally the BMI increased by 0.49. For a boy of average age, the longitudinal increase in BMI was estimated to be 0.58 per year. For older children, the longitudinal increase in BMI was smaller by 0.11 per year than the average. Similarly, the longitudinal increase in BMI for girls was significantly lower by 0.18. Both the level and slope variances were significant, showing that children reliably differed both in BMI at average age and, more importantly, in BMI increase over time. The significant level‐slope correlation was *r* = 0.35, implying that those who started out with a higher BMI tended to increase more in BMI over time.

**TABLE 2:** Parameter estimates from Model 0, Model 1, and Model 2.

	Model 0	Model 1	Model 2
Fixed effects			
Level	16.81*	16.81*	16.83*
Slope	0.58*	0.58*	0.50*
Age × level	0.49*	0.47*	0.46*
Sex × level	0.04	0.04	0.02
Edu^a^ × level		−0.32*	−0.25*
Job^b^ × level		−0.28*	−0.25*
TV^c^ × level			0.61*
Comp^d^ × level			−0.01
PA^e^ × level			−0.50*
Age × slope	−0.11*	−0.12*	−0.11*
Sex × slope	−0.18*	−0.18*	−0.20*
Edu^a^ × slope		−0.08*	−0.06
Job^b^ × level		0.00	0.00
TV^c^ × slope			0.15*
Comp^d^ × slope			−0.03
PA^e^ × slope			−0.14*
Within‐person effects			
TV^c^			0.23*
Comp^d^			0.04
PA^e^			−0.14*
Random effects			
Level variance	5.69*	5.40*	5.25
Slope variance	0.46*	0.42	0.45*
Level‐slope corr.	0.35*	0.32*	0.22
Model fit			
AIC	7071	7027	6956

^a^ Edu = parental education.

^b^ Job = parental job status.

^c^ TV = hours of watching TV or DVD per day.

^d^ Comp = hours of using the computer/Internet per day.

^e^ PA = hours of physical activities per day.

*p < 0.05.

In Model 1, parental education and job status (Level 2 variables) were added as predictor variables (see Table 2). A higher parental education significantly predicted a lower cross‐sectional BMI. Similarly, a better parental job status predicted a lower cross‐sectional BMI. No effects of parental education or job status on longitudinal BMI change were found.

**FIGURE 1:**
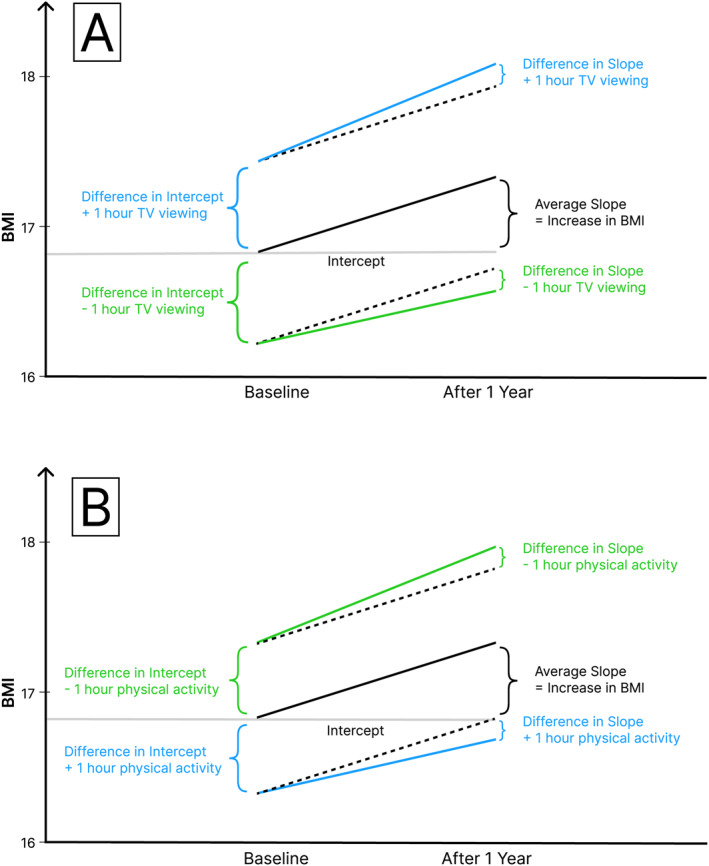
Parameter estimates from Model 2: cross‐sectional effects of hours of TV viewing and physical activity on BMI and BMI changes in children. Panel (A) depicts the cross‐sectional effects of daily hours of TV viewing. Panel (B) depicts the cross‐sectional effects of daily hours of physical activity.

**FIGURE 2:**
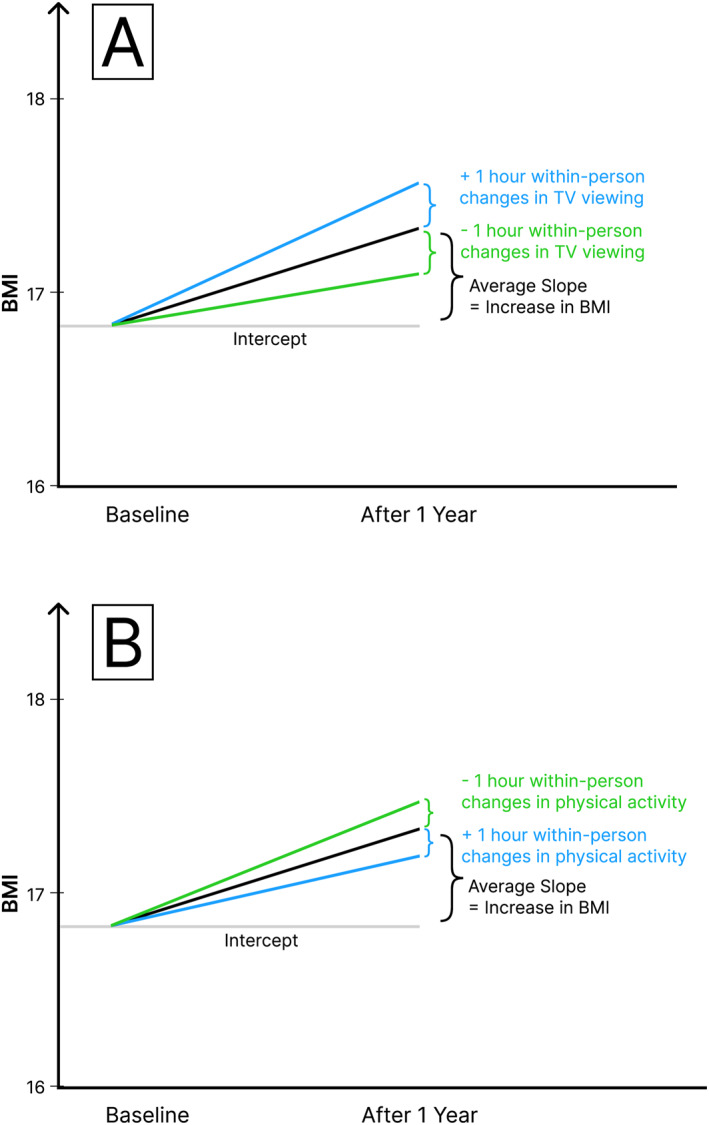
Parameter estimates from Model 2: within‐person effects of hours of TV viewing and physical activity on BMI changes in children. Panel (A) depicts the within‐person effects of daily hours of TV viewing. Panel (B) depicts the with‐person effects of daily hours of physical activity.

In Model 2, TV watching, computer time, and physical activity were included (see Table 2). Cross‐sectionally, hours of TV viewing predicted a higher BMI (0.61), whereas hours of physical activity predicted a lower BMI (−0.50). Similarly, longitudinally, hours of TV viewing predicted a significantly increased slope in BMI (0.15), implying that those who consumed more TV had a greater rise in BMI per year, whereas hours of physical activity predicted a significantly decreased slope in BMI (−0.14). In addition, hours of TV viewing and physical activity also showed significant within‐person effects (TV: 0.23; physical activity: −0.14). Specifically, this implies that within children, a change in hours of TV viewing between the two measurement occasions was accompanied by a corresponding change in BMI in the same direction. Conversely, a change in hours of physical activity between the two measurement occasions was related to a corresponding change in BMI in the opposite direction. No effects were found for hours of computer/Internet use per day. Model 2 also provided estimates of within‐person correlations between the predictor variables. Within a child, changes in hours of daily TV consumption were weakly but positively associated with changes in hours of physical activity (*r*[1940] = 0.13, *p* < 0.001). The same pattern was found for changes in hours of daily computer usage and changes in physical activity (*r*[1940] = 0.10, *p* < 0.001). Within‐person changes in hours of daily TV watching and daily computer use were also positively correlated (*r*[1940] = 0.34, *p* < 0.001). Parameter estimates from Model 2 were used to graphically show how hours of TV watching and hours of physical activity were related to BMI and BMI change (see Figures 1 and 2).

## 4 Discussion

The present study found that screen time was positively related to BMI cross‐sectionally, longitudinally and within‐person in a diverse sample of German children between the ages of 6–11. Specifically, at the cross‐sectional level, more hours of daily TV watching were associated with a higher BMI. In addition, more hours of daily TV watching were related to a greater increase in BMI from T1 to T2. Finally, within a child, an increase in hours of daily TV watching from the first to the second measurement occasion was positively related to a concurrent rise in BMI. Importantly, no effects of daily hours of computer or Internet use were found on children's BMI, either cross‐sectionally, longitudinally or within‐person. Therefore, the first hypothesis that an increase in screen time is associated with a rise in BMI in children was only supported for daily hours of TV viewing. This, however, is in line with previous research. For instance, in a review of prospective studies, only hours of TV consumption, but not hours of using the computer, were related to childhood overweight and obesity [42]. The authors suggested that this may be because TV consumption, but not using the computer, is associated with unhealthy eating habits and increased snack consumption. As an alternative explanation, they suggested that children may not spend as much time using computers or the Internet as they do watching TV. In the present sample, as reported by their parents, more than 80% of the children either never used computers or the Internet or used them for less than an hour a day, which may also explain the lack of effect of hours of computer or Internet use on children's BMI.

Another mechanism that has been proposed to promote increased body weight as a result of increased screen time in children is a reduction in physical activity [20]. Similar to TV viewing, in the present study, physical activity was associated with children's BMI cross‐sectionally, longitudinally, and within‐person, but in the opposite direction. Specifically, more hours of daily physical activity were associated with a lower BMI cross‐sectionally, and a smaller increase in BMI between T1 and T2. Within children, an increase in daily physical activity between T1 and T2 was negatively associated with a rise in BMI. The second hypothesis of the present study, that within a child, changes in physical activity would be negatively related to changes in BMI, was therefore fully supported by the present data.

Finally, the present study sought to investigate how within‐person changes in screen time were associated with within‐person changes in physical activity. Surprisingly, there was a small positive correlation between changes in both hours of TV consumption and hours of using the computer with physical activity. This finding is in line with studies that have argued that a reduction in TV viewing does not necessarily lead to an increase in physical activity [25], but conversely, it has often been argued that an increase in TV viewing should lead to a decrease in physical activity, one reason being time displacement [25, 43]. Notably, the present study found a small positive correlation between changes in screen time and physical activity, which argues against the time displacement theory in children. Children generally have more leisure time than adolescents and adults, and they spend it in a variety of different activities, such as reading or playing outside, only three of which were examined in the present study. The finding that within‐person increases (or decreases) in screen time are associated with increases (or decreases) in physical activity may be a consequence of the longitudinal design, as older children generally tend to have more screen time [44] compared to other activities such as quiet play, but are also more likely to be part of a sports group, for instance. Nevertheless, the present finding that changes in TV watching and physical activity are slightly positively correlated suggests that their effects on children's BMI are relatively independent, and in particular that the effects of TV viewing on children's BMI are mainly due to mechanisms other than reduced physical activity, such as unhealthy eating.

Importantly, beyond the effects of predictors, it was found that those children who started out with a relatively higher compared to lower BMI at baseline had a stronger increase in BMI between the two measurement occasions. Similarly, a longitudinal study showed that those children who were overweight at baseline had an 18 to 20 times greater chance of having obesity 7 years later than those with a healthy weight status at baseline [45]. This finding underlines the importance of targeting overweight and obesity already in childhood, as it represents a major risk factor for overweight, obesity and related health problems also in adulthood [3].

A limitation of the present study is that screen time was measured by hours of daily TV consumption and hours of daily computer/Internet usage but did not include smartphone use and daily hours spent scrolling through social media. The data for the present study were collected in 2012, a decade when smartphone and social media use were less prevalent. As has been previously demonstrated, problematic social media use is related to increased weight status in children and adolescents [46]. Importantly, however, TV viewing and social media use have been found to contribute to children's body weight through different mechanisms. TV viewing has been argued to increase unhealthy eating, while problematic social media use has been found to influence body weight through, for example, levels of life satisfaction, family communication [46], or the experience of stigmatization [47, 48]. Although smartphone and social media use has increased dramatically over the past decade, TV viewing remains common. Specifically, there is an increasing accessibility of movies and series through a growing number of streaming services, and the ability to watch them on a variety of different devices at any time, which is why the present results are still very relevant today. Therefore, future studies should further investigate the health effects of smartphone and social media use among children and adolescents.

Another limitation of the present study is that we only investigated changes in screen time, physical activity and BMI over the course of approximately 1 year in pre‐adolescent children. It would have been even more informative if there had been more than two measurement occasions over more than only 1 year in a wider age range, especially as screen time has been found to increase throughout adolescence [44].

Finally, future studies should investigate the mechanisms by which reductions in screen time lead to reductions in BMI in children and adolescents. A meta‐analysis of randomized controlled trials revealed that different interventions to reduce screen time, such as electronic time monitors or financial rewards for reduced screen time, were effective in reducing screen time but not in reducing BMI [19]. In contrast, the present observational study found that changes in screen time over the course of 1 year were related to changes in BMI. However, the present study did not test the effects of interventions on BMI in children but rather reported how observed fluctuations in screen time and physical activity were related to concurrent changes in BMI. Therefore, the present results should only be compared with those from intervention studies with caution. More research is needed to gain a deeper understanding of the association between BMI and screen time and the mechanisms involved in order to make concrete predictions for future interventions. However, what can be taken from the present findings is that TV consumption and physical activity appear to affect children's body weight through different mechanisms, even in a community sample with only low proportions of children with overweight or obesity. Typically, interventions in practice to reduce and prevent childhood overweight and obesity already focus on both, reducing daily TV viewing and promoting physical activity [49]. Therefore, the present findings further underline the importance of doing so.

## 5 Conclusion

The present study provides further evidence of the association between BMI and screen time in children. In particular, it has been shown that, longitudinally, an increase in hours of daily TV viewing within a child is related to a rise in BMI in the same child. It was also shown that within a child, a change in hours of daily physical activity was related to an opposite change in BMI. Surprisingly, changes in screen time were not related to changes in physical activity, suggesting that the effects of TV viewing on BMI mainly work through mechanisms other than physical activity, such as unhealthy eating. Future studies should further investigate the context in which a reduction in screen time leads to a reduction in BMI. Eventually, the present findings suggest that interventions to reduce and prevent childhood overweight and obesity should focus on both reducing daily TV viewing and promoting physical activity. In the current era, where technologies and access to them are developing rapidly while the rates of childhood overweight and obesity are increasing, clearer guidance on appropriate use of technologies, such as limiting of screen time, is needed, as well as a better understanding of their potential effects on physical and mental health, not only in children but across the lifespan.

## Conflicts of Interest

The authors declare no conflicts of interest.

## Data Availability

The raw data that support the findings of the present study will be made available by Petra Warschburger upon reasonable request.
